# Synergistic effect of chronic kidney disease, neuropathy, and retinopathy on all-cause mortality in type 1 and type 2 diabetes: a 21-year longitudinal study

**DOI:** 10.1186/s12933-022-01675-6

**Published:** 2022-11-05

**Authors:** Luca Sacchetta, Martina Chiriacò, Lorenzo Nesti, Simone Leonetti, Giovanna Forotti, Andrea Natali, Anna Solini, Domenico Tricò

**Affiliations:** 1 Laboratory of Metabolism, Nutrition, and Atherosclerosis, University of Pisa, Pisa, Italy; 2 Department of Clinical and Experimental Medicine, University of Pisa, Via Roma 67, 56126 Pisa, Italy; 3 Unit of Internal Medicine 5, University Hospital of Pisa, Pisa, Italy; 4 Department of Surgical, Medical and Molecular Pathology and Critical Care Medicine, University of Pisa, Via Savi 10, 56124 Pisa, Italy

**Keywords:** Diabetes mellitus, Microvascular complications, Renal dynamic scintigraphy, Diabetic kidney disease, Cardiac autonomic neuropathy, Diabetic retinopathy

## Abstract

**Background:**

The prognostic value of common and frequently associated diabetic microvascular complications (MVC), namely chronic kidney disease (CKD), cardiac autonomic neuropathy (CAN), peripheral neuropathy (DPN), and retinopathy (DR), is well established. However, the impact of their different combinations on long-term mortality has not been adequately assessed.

**Methods:**

We retrospectively analyzed 21-year longitudinal data from 303 patients with long-standing type 1 (T1D) or type 2 diabetes (T2D), who were thoroughly characterized at baseline for the presence of MVC using ^99m^Tc-DTPA dynamic renal scintigraphy, overnight urine collection, cardiovascular autonomic tests, monofilament testing, and dilated fundus oculi examination.

**Results:**

After a 5,244 person-years follow-up, a total of 133 (43.9%) deaths occurred. The presence of CKD and CAN, regardless of other MVC, increased the adjusted all-cause mortality risk by 117% (HR 2.17 [1.45–3.26]) and 54% (HR 1.54 [1.01–2.36]), respectively. Concomitant CKD&CAN at baseline were associated with the highest mortality risk (HR 5.08 [2.52–10.26]), followed by CKD&DR (HR 2.95 [1.63–5.32]), and CAN&DR (HR 2.07 [1.11–3.85]). Compared with patients free from MVC, the mortality risk was only numerically higher in those with any isolated MVC (HR 1.52 [0.87–2.67]), while increased by 203% (HR 3.03 [1.62–5.68]) and 692% (HR 7.92 [2.93–21.37]) in patients with two and three concomitant MVC, respectively.

**Conclusions:**

Our study demonstrates the long-term, synergistic, negative effects of single and concomitant diabetic MVC on all-cause mortality, which should encourage comprehensive screenings for MCV in both T1D and T2D to improve risk stratification and treatment.

**Supplementary Information:**

The online version contains supplementary material available at 10.1186/s12933-022-01675-6.

## Introduction

Chronic exposure to hyperglycemia in patients with diabetes mellitus impairs microvascular functions, frequently leading to microvascular complications (MVC). Diabetes related MVC share a common pathophysiology and are often associated in the same individual, posing a significant burden on both the healthcare systems and the patients. Among MVC, chronic kidney disease (CKD) has an estimated prevalence of 20–50% in type 1 diabetes (T1D) and type 2 diabetes (T2D) and is the leading cause of end-stage kidney disease (ESKD) [1]. Diabetic neuropathy, with an estimated prevalence of 6% to 51%, refers to a heterogeneous group of medical conditions, including cardiovascular autonomic neuropathy (CAN) and diabetic peripheral neuropathy (DPN), which can be complicated by cardiovascular disease (CVD) and lower-limb disease [2]. Diabetic retinopathy (DR) affects a third of patients with diabetes [3] and is the most common cause of vision loss in working-age individuals [4].

Epidemiological studies indicate that diabetes related MVC can increase the risk of incident CVD and all-cause mortality [2, 5–7]. Nonetheless, the cumulative impact of the different types of MVC on life expectancy has not been adequately explored. Most of the available studies focused on the prognostic role of single, isolated MVC, overlooking the potential interaction with concomitant conditions. Furthermore, studies are often limited to patients with T1D and characterized by short follow-up periods. An extended follow-up beyond 10 years is fundamental to appreciate late time-dependent effects, given the increased life expectancy of people with diabetes secondary to improved standards of care [8].

In this study, we examined the long-term prognostic role of diabetes related MVC, alone or in combinations, on all-cause mortality in both T2D and T1D. For this purpose, we retrospectively analyzed longitudinal data of a well-characterized cohort of patients with long-standing diabetes who underwent an extensive screening for the presence of MCV in 1999–2000 and were followed up for more than 20 years.

## Methods

### Study protocol

The “*CHronic diabetes complications and All-cause Mortality in Pisa from 1999 ONwards*” (CHAMP1ON) study is a single-center, observational study involving 497 consecutive outpatients referred to the University Hospital of Pisa for dysglycemia between 1999 and 2000 [9]. Inclusion criteria were age between 18 and 75 years, both women and men, history of diabetes or prediabetes (either impaired fasting glucose or impaired glucose tolerance). Most patients presented at baseline with comorbidities commonly associated with diabetes, including obesity and dyslipidemia. Exclusion criteria were concomitant acute or chronic diseases associated with a reduction in life expectancy, including ESKD, lung, hepatic, neoplastic or inflammatory diseases, and CVD events in the previous 12 months. At enrollment, all participants underwent a physical examination by a trained physician and a full clinical history was obtained. Patients were extensively characterized via biochemical and clinical exams and were thoroughly screened for the presence of major MVC, namely CKD, CAN, DPN, and DR.

**Fig. 1:**
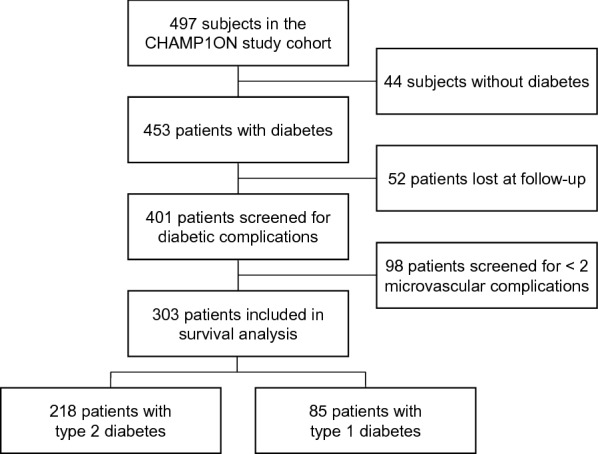
Flow diagram of patients’ selection

After enrollment, participants periodically attended the clinic in relation to their clinical needs and were treated according to the best clinical practice relevant to that time for the control of major cardiovascular risk factors. The vital status of study participants was retrieved in April 2021 from the Italian Health Care database, which provides updated information on current Italian residents. In this retrospective longitudinal study, we analyzed data of CHAMP1ON study participants with diabetes who were screened for at least two MVC and had available survival data (Fig. 1).

The study was approved by the local Human Ethics Committee and conducted in accordance with the principles expressed in the Declaration of Helsinki. All subjects provided written informed consent prior to enrollment.

### Assessment of diabetes MVC

The measured glomerular filtration rate (mGFR) was determined using dynamic renal scintigraphy with ^99m^Tc-DTPA, a glomerular-specific radiotracer [10]. The estimated glomerular filtration rate (eGFR) was calculated using the CKD-EPI creatinine equation [11]. Urinary albumin excretion was measured via radioimmunoassay using timed overnight urine collection, excluding samples that were indicative of significant urinary tract infection or hematuria. CKD was defined as a reduced mGFR (< 60 ml/min/1.73 m^2^) and/or microalbuminuria (20–200 µg/min) or macroalbuminuria (> 200 µg/min), in the absence of signs or symptoms of other primary causes of kidney damage [12].

A battery of validated cardiovascular tests for the assessment of heart rate variability (HRV) during lying-to-standing, standing-to-lying, and deep breathing was performed using a portable computerized system (Cardionomic, Medimatica, Martinsicuro, Italy) [13]. CAN was defined as at least two cardiovascular tests showing reduced HRV and/or orthostatic hypotension, defined as ≥ 20 mmHg reduction in SBP within 3 min of standing, according to the current diagnostic criteria [14]. A standardized questionnaire for symptoms of DPN, physical exam, and monofilament testing were used for the screening of DPN according to standard procedures [15]. If suspected, DPN was subsequently confirmed by electroneurography and electromyography.

Dilated fundus oculi examination was performed by a trained ophthalmologist using an ophthalmoscope. Pre-proliferative or proliferative DR was defined by the presence of any characteristic lesions, including intraretinal microvascular abnormalities, microaneurysms, hemorrhages, cotton wool spots, venous beading or dilation, hard exudates, and new vessels [16].

### Statistical analysis

Variables were tested for normality using the Shapiro–Wilk test. Continuous normally and non-normally distributed variables are presented as mean ± SD or median [interquartile range, or IQR], respectively. Categorical variables are presented as count (percentage). Overall survival (OS) was calculated as the time between the ascertained time of death and the date of enrollment. Kaplan–Meier curves were compared with the log-rank test. Cox proportional hazards models were used to estimate hazard ratios (HR) and 95% confidence intervals for all-cause mortality adjusted for age, sex, glycemic control (HbA1c), BMI, as well as duration and type of diabetes. Kaplan–Meier curves predicted by multivariate Cox regression models are shown. The effect of diabetes type was further examined by adding an interaction term between the type of diabetes and the variable of interest in all the adjusted models. Subgroup analyses by diabetes type were performed if a significant interaction was observed. The proportional hazards assumption was respected in all models. Group differences for continuous and categorical variables were tested using Mann–Whitney test or Fisher exact test, respectively. Statistical analysis was performed with JMP Pro software version 16 (SAS Institutes, Cary, NC) and STATA software version 16 (StataCorp, College Station, TX) at a two-sided α level of 0.05.

## Results

### Study participants

The study population consisted of 303 subjects, including 218 (71.9%) patients with T2D and 85 (28.1%) patients with T1D (Fig. 1). Demographic, clinical, and metabolic characteristics of study participants at enrolment are presented in Table 1. Women and men were evenly represented. Most patients had long diabetes duration (> 5 years, 71.6%) and suboptimal glucose control (HbA1c > 7.5%, 76.2%); one half was under insulin treatment (50.2%) and 15.8% were treated with lifestyle interventions only. Furthermore, most patients had overweight or obesity (BMI > 25 kg/m^2^, 68.6%), hypertension (55.9%) and hypercholesterolemia (LDL cholesterol > 100 mg/dl, 62.7%), and 16.2% were active smokers. At enrollment, 60.7% of patients had at least one MVC, and 31.3% of patients had 2 or more MVC. The prevalence was 29.7% for CKD, 28.3% for CAN, 21.1% for DPN, and 35.4% for DR.

**Table 1:** Baseline characteristics of study participants

Characteristics	
N	303
Age, years—median [IQR]	58 [19]
Women—no. (%)	155 (51.2)
Body weight, kg—median [IQR]	77 [19]
Body mass index, kg/m^2^—median [IQR]	27.7 [7.5]
Systolic blood pressure, mmHg—median [IQR]	140 [29]
Diastolic blood pressure, mmHg—median [IQR]	78 [11]
Diabetes mellitus—no. (%)	
Type 2 Diabetes—no. (%)	218 (71.9)
Type 1 Diabetes—no. (%)	85 (28.1)
Duration of diabetes—median [IQR]	11 [16]
Smoke—no. (%)	95 (31.4)
Hypertension—no. (%)	168 (55.4)
Fasting glucose, mg/dl—median [IQR]	166 [92]
HbA1c, %—median [IQR]	8.8 [2.7]
Total cholesterol, mg/dl—median [IQR]	211 [68]
HDL cholesterol, mg/dl—median [IQR]	45 [16]
LDL cholesterol, mg/dl—median [IQR]	132 [56]
Triglycerides, mg/dl—median [IQR]	142 [102]
Creatinine, mg/dl—median [IQR]	0.87 [0.26]
mGFR, ml/min/1.73 m^2^—median [IQR]	99.5 [37.3]
eGFR, ml/min/1.73 m^2^—median [IQR]	84.4 [26.3]
Albuminuria, μg/min—median [IQR]	7.6 [14.6]
Glucose-lowering therapy—no. (%)	255 (84.2)
Oral agents—no. (%)	117 (38.6)
Biguanides—no. (%)	128 (42.2)
Sulphonylureas—no. (%)	85 (28.1)
Acarbose—no. (%)	9 (3.0)
Insulin—no. (%)	152 (50.2)
Insulin, UI/die—median [IQR]	40 [18]
Lipid-lowering therapy—no. (%)	26 (8.6)
Anti-hypertensive therapy—no. (%)	168 (55.5)
ACEi/ARB—no. (%)	121 (39.9)
Beta-Blockers—no. (%)	18 (5.9)
Ca-Blockers—no. (%)	68 (22.4)
Alpha-Blockers—no. (%)	33 (10.9)

*ACEi* angiotensin converting enzyme inhibitors, *ARB* angiotensin II receptors blockers, *eGFR* estimated glomerular filtration rate, *HDL* high-density lipoprotein, *IQR* interquartile range, *LDL* low-density lipoprotein, *mGFR* measured glomerular filtration rate

### Long-term prognostic value of single MVC

**Fig. 2:**
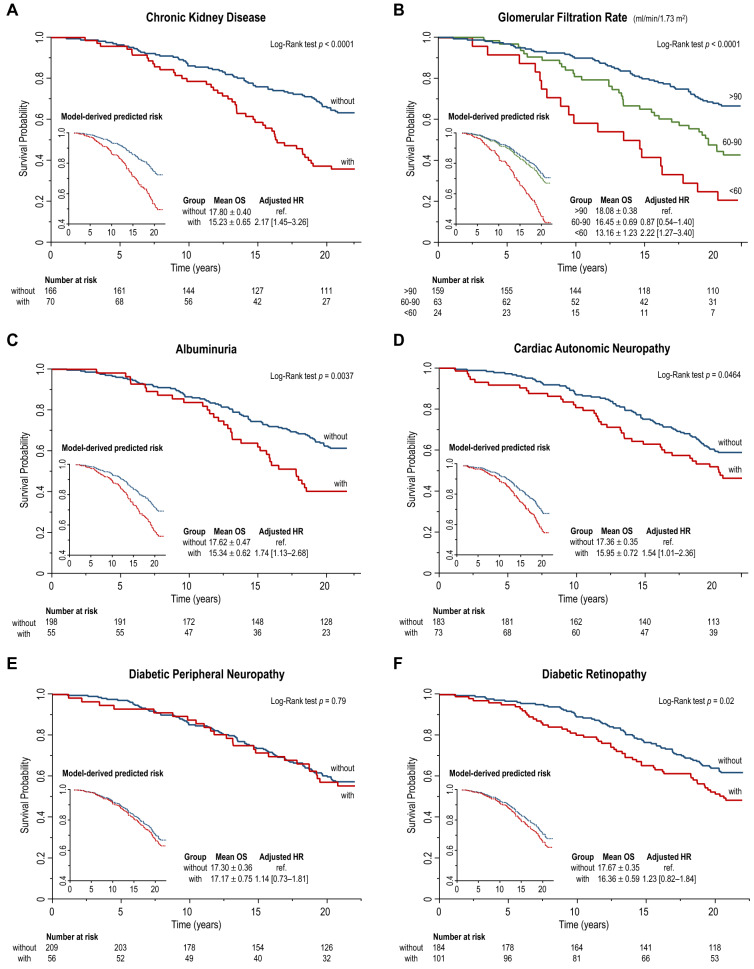
Kaplan–Meier and model-predicted survival curves for single diabetic microvascular complications, including chronic kidney disease (**A**) and its components, namely reduced glomerular filtration rate (**B**) and albuminuria (**C**), cardiac autonomic neuropathy (**D**), diabetic peripheral neuropathy (**E**), and diabetic retinopathy (**F**)

After 5,244 person-years of follow-up (median follow-up 21.0 [IQR 6.7] years), a total of 133 (43.9%) deaths occurred. Kaplan–Meier and model-predicted survival curves for each single MVC, regardless of the presence of other MCV, are shown in Fig. 2.

The presence of CKD reduced mean OS by 2.6 years (− 14.4%; log-rank test *p* < 0.0001) and increased the mortality risk by 117% in the adjusted model (HR 2.17 [1.45–3.26]) (Fig. 2A). Consistently, mean OS was reduced by 1.6 years (− 9.0%) in patients with mGFR between 60 and 90 ml/min/1.73 m^2^, and by 4.9 years (− 27.2%) in patients with mGFR < 60 ml/min/1.73 m^2^, compared with patients with mGFR > 90 ml/min/1.73 m^2^ (log-rank test *p* < 0.0001) (Fig. 2B). An mGFR < 60 ml/min/1.73 m^2^ was associated with a 122% increase in the adjusted risk for all-cause mortality (HR 2.22 [1.27–3.40]) compared with mGFR > 90 ml/min/1.73 m^2^, while there was no difference in the adjusted mortality risk between the groups with mGFR 60–90 ml/min/1.73 m^2^ and > 90 ml/min/1.73 m^2^. Patients with albuminuria (of whom 25.5% with macroalbuminuria) had a reduction in mean OS of 2.3 years (− 12.9%; log-rank test *p* = 0.004) and a 74% increase in all-cause mortality risk in the adjusted model (HR 1.74 [1.13–2.68]) (Fig. 2C).

Patients with CAN showed a reduction in mean OS of 1.4 years (− 8.1%; log-rank test *p* = 0.046) and a 54% increase in all-cause mortality risk in the adjusted model (HR 1.54 [1.01–2.36]) compared with those without CAN. In contrast, DPN was not associated with OS reduction (log-rank test *p* = 0.79) or adjusted mortality risk (HR 1.14 [0.73–1.81]).

The presence of DR reduced mean OS by 1.3 years (− 7.4%; log-rank test *p* = 0.02) (Fig. 2D). The adjusted risk for all-cause mortality in patients with DR was numerically higher, albeit not statistically different, than in those without DR (HR 1.23 [0.82–1.84]). This result was confirmed in secondary analyses adjusted by mean systolic blood pressure and hypertension.

When testing for the influence of diabetes type on the prognostic role of each MVC, a significant interaction was found for CAN only (*p* = 0.043), while CKD, DPN, and DR had similar effects in both T2D and T1D. In subgroup analyses by type of diabetes, CAN increased the adjusted mortality risk in patients with T2D (HR 1.78 [1.13–2.81]) but not in patients with T1D (HR 0.98 [0.30–3.18]).

### Long-term prognostic value of different MVC pairs

**Fig. 3:**
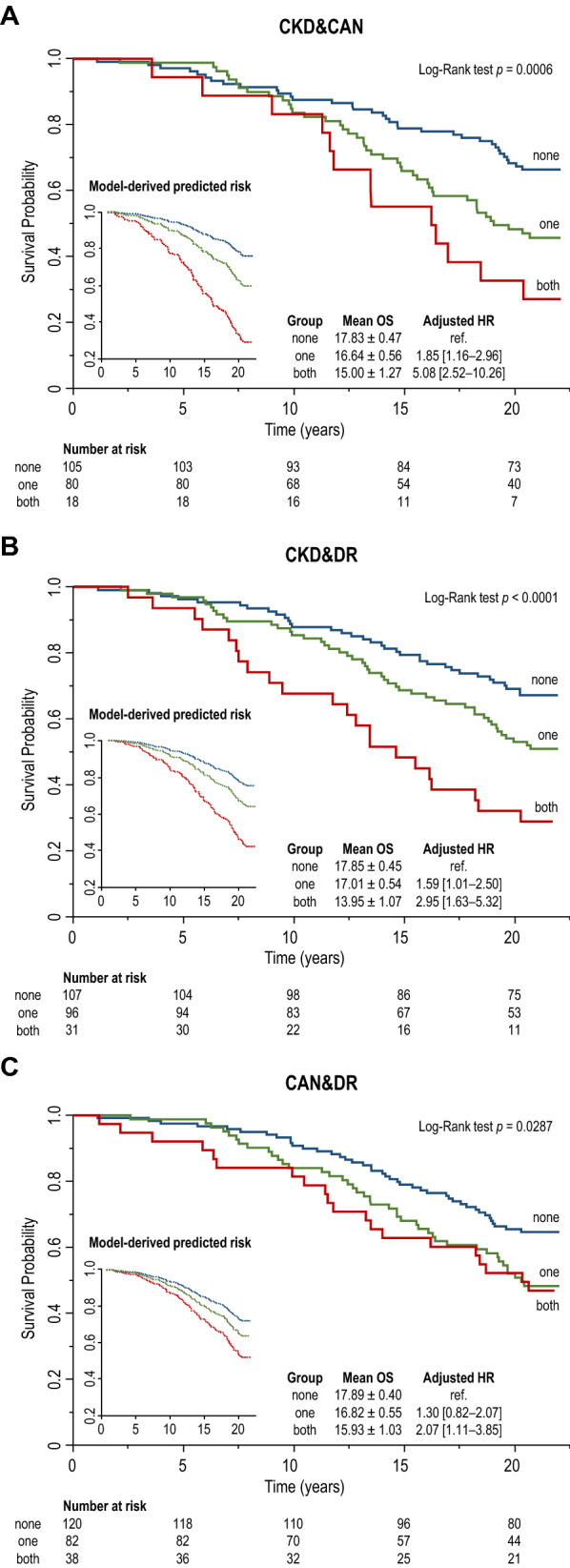
Kaplan–Meier and model-predicted survival curves for pairwise combinations of diabetic microvascular complications, including chronic kidney disease (CKD) and cardiac autonomic neuropathy (CAN) (**A**), CKD and diabetic retinopathy (DR) (**B**), and CAN&DR (**C**)

Kaplan–Meier and model-predicted survival curves for the different pairwise combinations of MVC associated with reduced life expectancy in our cohort are shown in Fig. 3.

Concomitant CKD and CAN were associated with a reduction of 2.83 years in mean OS (− 15.9%; log-rank test *p* = 0.0006) and with the greatest increase in the adjusted risk for all-cause mortality (HR 5.08 [2.52–10.26]), compared with patients free from CKD and CAN (Fig. 3A). The presence of both CKD and DR was associated with the greatest reduction in mean OS (3.90 years or − 21.9%; log-rank test *p* < 0.0001) and a 195% increase in the adjusted mortality risk (HR 2.95 [1.63–5.32]) (Fig. 3B). Finally, patients with concomitant CAN and DR showed a 1.96-year (− 11.0%) reduction in mean OS (log-rank test *p* = 0.03) and 107% increased risk of all-cause mortality (HR 2.07 [1.11–3.85]) (Fig. 3C).

### Long-term prognostic value of multiple concomitant MVC

**Fig. 4:**
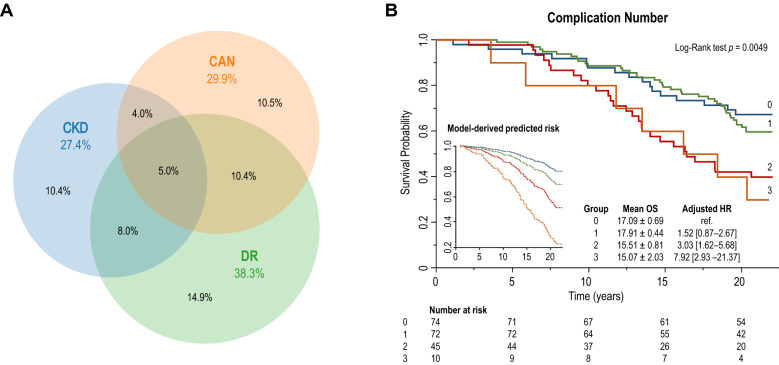
Distribution of diabetic microvascular complications in patients characterized for the presence of chronic kidney disease (CKD), cardiac autonomic neuropathy (CAN), and diabetic retinopathy (DR) (**A**). Kaplan–Meier and model-predicted survival curves for the total number of concomitant diabetic microvascular complications among CKD, CAN, and DR (**B**)

The subgroup of patients fully characterized for the presence of CKD, CAN, and DR had similar characteristics (Additional file 1: Table S1) and MCV distribution (Fig. 4A) compared with the whole study cohort. Baseline characteristics of these subjects stratified by the number of MVC are summarized in Additional file 1: Table S2. Kaplan–Meier and model-predicted survival curves for the total number of MVC among CKD, CAN, and DR are shown in Fig. 4B.

Compared with patients without any MVC, patients with one, two, or three MVC showed a progressive reduction in mean OS of 0.48 years (− 2.7%), 2.52 years (− 14.0%), and 2.96 years (− 16.4%), respectively (log-rank test *p* = 0.0016). The adjusted risk for all-cause mortality was numerically higher in patients with any isolated MVC compared with those without MVC (HR 1.52 [0.87–2.67]), and increased by 203% (HR 3.03 [1.62–5.68]) and 692% (HR 7.92 [2.93–21.37]) in patients with two or three concomitant MVC, respectively.

## Discussion

In this study, we elucidated the long-term prognostic role of single and concomitant MVC in a cohort of middle-aged patients with long-standing T1D or T2D. Over a remarkably long follow-up period of 21 years, CKD, CAN, and DR, but not DPN, reduced life expectancy by 1.3 to 2.6 years, and CKD and CAN increased the adjusted all-cause mortality by 117% and 54%, respectively. Importantly, the accurate assessment of multiple concomitant MVC allowed us to establish the synergistic effect of their combination on all-cause mortality, which is of clinical relevance as diabetic MVC are frequently associated. The presence of both CKD and CAN markedly increased mortality risk by fivefold. Furthermore, their combination with DR was associated with an even greater reduction in survival and increased mortality risk, as compared with the presence of any isolated MVC. This novel evidence substantiates the clinical value of a comprehensive screening for isolated or combined MVC in patients with diabetes for better risk stratification.

Previous studies with indirect estimation of GFR (calculated eGFR) established the role of CKD on CVD-related and all-cause mortality risk, regardless of the presence of other complications, often over follow-up periods shorter than 5–10 years [17–22]. Although the estimation of GFR based on serum creatinine level is feasible and widely used in large-scale studies and clinical practice, numerous limitations are known in reflecting measured renal function. Indeed, eGFR may differ from mGFR by ± 30%, resulting in 30–60% misclassification of CKD stage [23]. Furthermore, creatinine-based eGFR formulae are adjusted for clinical factors that can directly affect mortality rate, including age, sex, and race, which would eventually lead to an over- or underestimation of the mortality risk associated with CKD. Our findings extend previous indirect evidence [17–22] confirming the prognostic value of GFR in diabetes using a direct measurement of GFR, based on ^99^Tc-DTPA renal dynamic scintigraphy, and exploiting a follow-up period of more than 20 years. This long observation period, approaching the life expectancy of a typical adult patient referred for complicated T2D, is apt to appreciate the full magnitude of the time-dependent, negative prognostic impact of MVC. This is particularly important for CKD and its components, whose effects on mortality risk become increasingly evident after 5 to 10 years of observation (Fig. 2A–C).

Diabetic neuropathy is a frequent but often overlooked MVC associated with CVD and mortality [24–34]. In a recent meta-analysis [35], CAN demonstrated a pooled relative risk of all-cause mortality of 3.17 [2.11–4.78], which was higher in T1D than T2D. In the 19 available studies identified, however, the follow-up length ranged widely (up to 21 years in DCCT/EDIC study participants with T1D [31]) and the number of abnormal autonomic function tests used to define CAN varied from 1 to 3, thus making results difficult to compare, especially for the T2D subgroup [35]. In our analyses, CAN was defined by applying newer diagnostic criteria [14], which reduce the risk of overestimating the incidence of CAN, and its negative prognostic role was reappraised in both T2D and T1D over an extended follow-up period. In contrast to previous observations, in our cohort CAN increased the mortality risk preferentially in T2D, even though patients with T1D showed similar CAN prevalence and severity. This finding should be interpreted with caution, as it may depend on several unaccounted factors, including different disease duration or therapeutic management in the two groups, and on the limited number of participants with T1D and CAN in our cohort. The same considerations, along with the low prevalence of complicated DPN (*e.g.* foot ulcers), may explain our negative result on the prognostic value of DPN [28, 32–34, 34].

It has been reported that individuals with DR have a greater incidence of CVD and lower survival rates than those without DR [36–40]. In the meta-analysis by Kramer et al. [39], the presence of DR was linked to a markedly increased risk for all-cause mortality in both T2D (OR 2.41 [1.87–3.10]) and T1D (OR 3.65 [1.05–12.66]). Our findings are partially in agreement with these results, as we observed an increase in the relative mortality risk associated with DR, which, however, lost statistical significance after accounting for potential confounders. This observation is consistent with two recent studies in large cohorts of patients with T2D [41] and T1D [42] showing that DR has a neutral effect on mortality independently of known confounders. Nonetheless, our negative findings can derive as well from peculiar participants’ characteristics and limited statistical power to assess the independent effect of DR in our cohort.

Multiple diseases with a common pathophysiology may compete for determining negative health outcomes, resulting in less-than-additive effects. In contrast, sparse evidence indicates that having two or more concomitant MVC may result in an additive or even synergistic effect on incident CVD and survival rates, compared with having a single isolated MVC, particularly in T1D [42–45]. In fact, CAN [30] and DR [46] were independent predictors of mortality only in T1D patients with CKD, but not in those without CKD, in two 5- to 10-year longitudinal studies. Consistently, we observed a steep increase in mortality rates when CAN and/or DR were associated with CKD, extending previous observations to patients with both T1D and T2D on a longer follow up.

Study participants presented with poor metabolic control, high prevalence of MCV (especially DR), and were naïve from any novel disease-modifier treatment. In fact, therapeutic targets of traditional risk factors in the late nineties were not as strict as today, and referral of patients with diabetes to a tertiary level clinic was often delayed. Also, the two classes of drugs with glucose-independent protective effects against diabetic MVC, namely glucagon-like peptide 1 (GLP-1) receptor agonists [47] and sodium-glucose co-transporter 2 (SGLT-2) inhibitors [48], were approved in T2D in 2005 and 2013, respectively, and became widely used only later. This partly justifies the high mortality rate in our cohort with respect to current life expectancy, being in line with studies performed in the same years [49]. It also underscores the significant ameliorations in quality of life and survival secondary to the recent advances in diabetes research and clinical practice, confirming that an early and vigorous control of glycemia and cardiovascular risk factors can deeply affect survival in diabetes, which would be otherwise poor when MVC arise.

There are some limitations to the present study, mostly related to its retrospective design. First, we could not retrieve reliable information on incident CVD events or the cause of death. Although MVC are stronger risk factors for CVD mortality than for non-CVD mortality [28], this precluded us to detail the prognostic role of each MVC on relevant clinical outcomes. Second, we could not evaluate the time course of MVC, clinical parameters including glycemic control, nor treatment changes after enrollment, which may influence study results. Thus, the prognostic role of the temporal association between different MVC and the potential effects of time-changing unaccounted factors should be assessed in future studies. Third, the number of subjects and events in some analyses was limited, especially when dealing with the T1D subgroup, which warrants caution in the interpretation of negative findings. Forth, the diagnosis of DR relied on a single operator-dependent assessment, which however was performed by a trained ophthalmologist to minimize the risk of misclassifications.

In conclusion, our study demonstrates the long-term, synergistic, negative effects of diabetes MVC on all-cause mortality in patients with both T2D and T1D. The substantial burden of MVC in diabetes fosters a comprehensive baseline screening to identify the subgroup of patients with higher mortality risk, who may benefit the most from targeted interventions to improve survival and quality of life.

## Supplementary Information

**Additional file 1:** **Table S1. **Baseline characteristics of the subgroup of study participants fully characterized for the presence of CKD, CAN, and DR. **Table S2. **Baseline characteristics of the study participants fully characterized for the presence of CKD, CAN, and DR stratified by the number of MVC.

## Data Availability

The data that support the findings of this study are available from the corresponding author upon reasonable request.

## Abbreviations

CAN: Cardiac autonomic neuropathy
CKD: Chronic kidney disease
DPN: Diabetic peripheral neuropathy
DR: Diabetic retinopathy
ESKD: End-stage kidney disease
GFR: Glomerular filtration rate
MVC: Microvascular complication
OS: Overall survival
T2D: Type 2 diabetes mellitus
T1D: Type 1 diabetes mellitus

## References

[1] Thomas MC (2015). Diabetic kidney disease. Nat Rev Dis Primers.

[2] Faselis C (2020). Microvascular complications of type 2 diabetes mellitus. Curr Vasc Pharmacol.

[3] Cheung N, Mitchell P, Wong TY (2010). Diabetic retinopathy. The Lancet.

[4] Mohamed Q, Gillies MC, Wong TY (2007). Management of diabetic retinopathy. JAMA.

[5] Fisher VL, Tahrani AA (2017). Cardiac autonomic neuropathy in patients with diabetes mellitus: current perspectives. Diabetes Metab Syndr Obes.

[6] de Zeeuw D, Parving HH, Henning RH (2006). Microalbuminuria as an early marker for cardiovascular disease. J Am Soc Nephrol.

[7] Penno G (2011). Clinical significance of nonalbuminuric renal impairment in type 2 diabetes. J Hypertens.

[8] Kianmehr H (2022). Potential gains in life expectancy associated with achieving treatment goals in US adults with type 2 diabetes. JAMA Network Open.

[9] Chiriaco M (2022). Prognostic value of 24-hour ambulatory blood pressure patterns in diabetes: a 21-year longitudinal study. Diabetes Obes Metab.

[10] Rehling M (1984). Simultaneous measurement of renal clearance and plasma clearance of 99mTc-labelled diethylenetriaminepenta-acetate, 51Cr-labelled ethylenediaminetetra-acetate and inulin in man. Clin Sci (Lond).

[11] Levey AS (2009). A new equation to estimate glomerular filtration rate. Ann Intern Med.

[12] American Diabetes Association Professional Practice (2022). Chronic kidney disease and risk management: standards of medical care in diabetes-2022. Diabetes Care.

[13] Vespasiani G (1996). Validation of a computerised measurement system for guided routine evaluation of cardiovascular autonomic neuropathy. Comput Methods Programs Biomed.

[14] Spallone V (2011). Cardiovascular autonomic neuropathy in diabetes: clinical impact, assessment, diagnosis, and management. Diabetes Metab Res Rev.

[15] Mueller MJ (1996). Identifying patients with diabetes mellitus who are at risk for lower-extremity complications: use of semmes-weinstein monofilaments. Phys Ther.

[16] Flaxel CJ (2020). Diabetic retinopathy preferred practice pattern(R). Ophthalmology.

[17] Kramer H (2018). Increasing mortality in adults with diabetes and low estimated glomerular filtration rate in the absence of albuminuria. Diabetes Care.

[18] Lees JS (2019). Glomerular filtration rate by differing measures, albuminuria and prediction of cardiovascular disease, mortality and end-stage kidney disease. Nat Med.

[19] Packham DK (2012). Relative incidence of ESRD versus cardiovascular mortality in proteinuric type 2 diabetes and nephropathy: results from the DIAMETRIC (Diabetes Mellitus Treatment for Renal Insufficiency Consortium) database. Am J Kidney Dis.

[20] Gerstein HC (2001). Albuminuria and risk of cardiovascular events, death, and heart failure in diabetic and nondiabetic individuals. JAMA.

[21] Chronic Kidney Disease Prognosis Consortium (2010). Association of estimated glomerular filtration rate and albuminuria with all-cause and cardiovascular mortality in general population cohorts: a collaborative meta-analysis. Lancet.

[22] Amin AP (2013). The Synergistic Relationship Between Estimated GFR and microalbuminuria in predicting long-term progression to ESRD or death in patients with diabetes: results from the Kidney Early Evaluation Program (KEEP). Am J Kidney Dis.

[23] Porrini E (2018). Estimated GFR: time for a critical appraisal. Nat Rev Nephrol.

[24] Gerritsen J (2001). Impaired autonomic function is associated with increased mortality, especially in subjects with diabetes, hypertension, or a history of cardiovascular disease: the Hoorn Study. Diabetes Care.

[25] Schonauer M (2008). Cardiac autonomic diabetic neuropathy. Diab Vasc Dis Res.

[26] Katz A (1999). A simple bedside test of 1-minute heart rate variability during deep breathing as a prognostic index after myocardial infarction. Am Heart J.

[27] Valensi P (2001). Predictive value of cardiac autonomic neuropathy in diabetic patients with or without silent myocardial ischemia. Diabetes Care.

[28] Soedamah-Muthu SS (2008). Relationship between risk factors and mortality in type 1 diabetic patients in Europe. Diabetes Care.

[29] Pop-Busui R (2010). Effects of cardiac autonomic dysfunction on mortality risk in the Action to Control Cardiovascular Risk in Diabetes (ACCORD) trial. Diabetes Care.

[30] Astrup AS (2006). Cardiac autonomic neuropathy predicts cardiovascular morbidity and mortality in type 1 diabetic patients with diabetic nephropathy. Diabetes Care.

[31] Pop-Busui R (2017). Cardiovascular autonomic neuropathy and cardiovascular outcomes in the diabetes control and complications Trial/Epidemiology of Diabetes Interventions and Complications (DCCT/EDIC) Study. Diabetes Care.

[32] Coppini DV (2000). Showing neuropathy is related to increased mortality in diabetic patients—a survival analysis using an accelerated failure time model. J Clin Epidemiol.

[33] Forsblom CM (1998). Risk factors for mortality in Type II (non-insulin-dependent) diabetes: evidence of a role for neuropathy and a protective effect of HLA-DR4. Diabetologia.

[34] Hicks CW (2021). Peripheral Neuropathy and all-cause and cardiovascular mortality in US Adults : a prospective cohort study. Ann Intern Med.

[35] Chowdhury M (2021). Cardiac autonomic neuropathy and risk of cardiovascular disease and mortality in type 1 and type 2 diabetes: a meta-analysis. BMJ Open Diabetes Research Care.

[36] Lovestam-Adrian M, Hansson-Lundblad C, Torffvit O (2007). Sight-threatening retinopathy is associated with lower mortality in type 2 diabetic subjects: a 10-year observation study. Diabetes Res Clin Pract.

[37] Xu XH (2020). Diabetic retinopathy predicts cardiovascular mortality in diabetes: a meta-analysis. BMC Cardiovasc Disord.

[38] Miettinen H (1996). Retinopathy predicts coronary heart disease events in NIDDM patients. Diabetes Care.

[39] Kramer CK (2011). Diabetic retinopathy predicts all-cause mortality and cardiovascular events in both type 1 and 2 diabetes. Diabetes Care.

[40] Xie J (2017). Association of diabetic macular edema and proliferative diabetic retinopathy with cardiovascular disease. JAMA Ophthalmology.

[41] Kaze AD (2021). Microvascular disease and cardiovascular outcomes among individuals with type 2 diabetes. Diabetes Research and Clinical Practice.

[42] Bjerg L (2019). Effect of duration and burden of microvascular complications on mortality rate in type 1 diabetes: an observational clinical cohort study. Diabetologia.

[43] Garofolo M (2019). Microvascular complications burden (nephropathy, retinopathy and peripheral polyneuropathy) affects risk of major vascular events and all-cause mortality in type 1 diabetes: a 10-year follow-up study. Cardiovasc Diabetol.

[44] Gordin D (2018). Differential association of microvascular attributions with cardiovascular disease in patients with long duration of type 1 diabetes. Diabetes Care.

[45] Brownrigg JRW (2016). Microvascular disease and risk of cardiovascular events among individuals with type 2 diabetes: a population-level cohort study. Lancet Diabetes Endocrinol.

[46] Pavkov ME (2019). Prevalence of diabetic retinopathy and associated mortality among diabetic adults with and without chronic kidney disease. Am J Ophthalmol.

[47] Trico D, Solini A (2021). Glucagon-like peptide-1 receptor agonists-use in clinical practice. Adv Chronic Kidney Dis.

[48] Chiriaco M, Trico D, Solini A (2022). Mechanisms of cardio-renal protection of sodium-glucose cotransporter-2 inhibitors. Curr Opin Pharmacol.

[49] Jensen MH (2019). Association of severe hypoglycemia with mortality for people with diabetes mellitus during a 20-year follow-up in Denmark: a cohort study. Acta Diabetol.

