# Supplementary material for: Synergistic effect of chronic kidney disease, neuropathy, and retinopathy on all-cause mortality in type 1 and type 2 diabetes: a 21-year longitudinal study
Source: Cardiovasc Diabetol. 2022 Nov 5;21:233. doi: 10.1186/s12933-022-01675-6 (PMC9637307; doi:10.1186/s12933-022-01675-6)
Supplement: Supplementary file 1 — Additional file 1: Table S1. Baseline characteristics of the subgroup of study participants fully characterized for the presence of CKD, CAN, and DR. Table S2. Baseline characteristics of the study participants fully characterized for the presence of CKD, CAN, and DR stratified by the number of MVC. [file 12933_2022_1675_MOESM1_ESM.pdf]

Supplemental Materials

Synergistic effect of chronic kidney disease, neuropathy, and retinopathy on all-cause mortality in type 1 and type 2 diabetes: a 21-year longitudinal study.

Luca Sacchetta[1,2]; Martina Chiriacò[1,2]; Lorenzo Nesti[1,3]; Simone Leonetti[1]; Giovanna Forotti[3]; Andrea Natali[1,2]; Anna Solini[4,*]; Domenico Tricò[1,2,*]

1. Laboratory of Metabolism, Nutrition, and Atherosclerosis, University of Pisa, Pisa, Italy
2. Department of Clinical and Experimental Medicine, University of Pisa, Pisa, Italy
3. Unit of Internal Medicine 5, University Hospital of Pisa
4. Department of Surgical, Medical and Molecular Pathology and Critical Care Medicine, University of Pisa, Pisa, Italy

* The last two authors share the last name

Corresponding authors:

- Domenico Tricò; Department of Clinical and Experimental Medicine, University of Pisa, Via Roma 67, 56126 Pisa, Italy; Tel: +39 050 993532, Fax: +39 050 553335; email: domenico.trico@unipi.it. ORCID: 0000-0002-7633-1346
- Anna Solini; Department of Surgical, Medical and Molecular Pathology and Critical Care Medicine, University of Pisa, Via Savi 10, 56124 Pisa, Ital; Tel: +39 050 993482; email: anna.solini@unipi.it. ORCID: 0000-0002-7855-8253

Supplemental Table 1. Baseline characteristics of the subgroup of study participants fully characterized for the presence of CKD, CAN, and DR.

**Characteristics**		***p ****
N	201	
Age, years – median [IQR]	58 [16]	*0.790*
Women – no. (%)	102 (50.8)	*0.928*
Body weight, kg – median [IQR]	77 [18]	*0.578*
Body mass index, kg/m2 – median [IQR]	27.7 [7.7]	*0.747*
Systolic blood pressure, mmHg – median [IQR]	137 [29.5]	*0.499*
Diastolic blood pressure, mmHg – median [IQR]	80 [12]	*0.327*
Diabetes mellitus – no. (%)		*0.589*
Type 2 Diabetes – no. (%)	52 (25.9)	
Type 1 Diabetes – no. (%)	149 (74.1)	
Duration of diabetes – median [IQR]	13 [15]	*0.377*
Smoke – no. (%)	60 (29.9)	*0.542*
Hypertension – no. (%)	108 (53.7)	*0.705*
Fasting glucose, mg/dl – median [IQR]	172 [100.5]	*0.421*
HbA1c, % – median [IQR]	9.1 [2.8]	*0.073*
Total cholesterol, mg/dl – median [IQR]	211 [69.5]	*0.909*
HDL cholesterol, mg/dl – median [IQR]	45 [13]	*0.745*
LDL cholesterol, mg/dl – median [IQR]	130 [62]	*0.899*
Triglycerides, mg/dl – median [IQR]	138 [95]	*0.944*
Creatinine, mg/dl – median [IQR]	0.88 [0.25]	*0.837*
mGFR, ml/min/1.73 m^2 – median [IQR]	100 [32]	*0.962*
eGFR, ml/min/1.73 m^2 – median [IQR]	85 [26.6]	*0.912*
Albuminuria, µg/min – median [IQR]	7.5 [14.2]	*0.905*
Glucose-lowering therapy – no. (%)	164 (81.6)	*0.468*
Oral agents – no. (%)	80 (39.8)	*0.518*
Insulin – no. (%)	107 (53.2)	*0.499*
Insulin, UI/die – median [IQR]	40 [18]	*0.839*
Lipid-lowering therapy – no. (%)	14 (7.0)	*0.618*
Anti-hypertensive therapy – no. (%)	108 (53.7)	*0.716*
ACEi/ARB – no. (%)	86 (46.5)	*0.446*
Beta-Blockers – no. (%)	9 (4.5)	*0.549*
Ca-Blockers – no. (%)	44 (21.9)	*0.868*
Alpha-Blockers – no. (%)	21 (10.5)	*0.968*

* For the difference with the whole study cohort.
Abbreviations: ACEi, angiotensin converting enzyme inhibitors; ARB, angiotensin II receptors blockers; CAN, cardiac autonomic neuropathy; CKD, chronic kidney disease; DR; diabetic retinopathy; eGFR, estimated glomerular filtration rate; HDL, high-density lipoprotein; IQR, interquartile range; LDL, low-density lipoprotein; mGFR, measured glomerular filtration rate.

Supplemental Table 2. Baseline characteristics of the study participants fully characterized for the presence of CKD, CAN, and DR stratified by the number of MVC.

**Characteristics**	**No MVC**	**1 MVC**	**≥ 2 MVC**	***p***
N (%)	74 (36.8)	72 (35.8)	55 (27.4)	
Age, years – median [IQR]	56 [18]	58 [18]	59 [13]	*0.350*
Women – no. (%)	34 (46.0)	33 (45.8)	26 (47.3)	*0.575*
Body weight, kg – median [IQR]	75.5 [17.7]	80.5 [20] [a]	75.6 [16.5]	*0.048*
Body mass index, kg/m2 – median [IQR]	26.2 [6.7]	29.2 [9.5] [a]	28.0 [5.7] [a]	*0.023*
Systolic blood pressure, mmHg – median [IQR]	130 [19]	140 [24] [a]	144 [32] [a]	*0.009*
Diastolic blood pressure, mmHg – median [IQR]	80 [9]	83 [12]	80 [16]	*0.146*
Diabetes mellitus – no. (%)				*0.124*
Type 2 Diabetes – no. (%)	16 (21.6)	16 (22.2)	20 (36.4)	
Type 1 Diabetes – no. (%)	58 (78.4)	56 (77.8)	35 (63.6)	
Duration of diabetes – median [IQR]	10 [12]	12 [16]	20 [16] [a, b]	*<0.001*
Smoke – no. (%)	25 (37.8)	20 (27.0)	15 (27.3)	*0.820*
Hypertension – no. (%)	29 (39.2)	41 (56.9)	38 (69.1)	*0.003*
Fasting glucose, mg/dl – median [IQR]	156.6 [89]	180 [39]	182 [120]	*0.171*
HbA1c, % – median [IQR]	8.6 [2.9]	9.3 [2.3]	9.5 [3.9] [a]	*0.024*
Total cholesterol, mg/dl – median [IQR]	216 [72]	218 [67]	203 [55]	*0.421*
HDL cholesterol, mg/dl – median [IQR]	44 [11]	46 [17]	45 [13]	*0.733*
LDL cholesterol, mg/dl – median [IQR]	133 [67]	131 [60]	128 [42]	*0.937*
Triglycerides, mg/dl – median [IQR]	143 [96]	136 [104]	135 [98]	*0.722*
Creatinine, mg/dl – median [IQR]	0.85 [0.23]	0.81 [0.28]	0.98 [0.34] [a, b]	*0.001*
mGFR, ml/min/1.73 m^2 – median [IQR]	107.0 [26.8]	98.0 [26.8] [a]	88.0 [51.0] [a, b]	*0.002*
eGFR, ml/min/1.73 m^2 – median [IQR]	89.9 [22.7]	85.6 [27.6]	74.7 [29.7] [a, b]	*0.002*
Albuminuria, µg/min – median [IQR]	5.0 [4.0]	8.8 [17.9] [a]	18.0 [45.9] [a, b]	*<0.001*
Glucose-lowering therapy – no. (%)	58 (78.4)	62 (86.1)	44 (80.0)	*0.443*
Oral agents – no. (%)	28 (37.8)	36 (50.0)	16 (29.1)	*0.090*
Insulin – no. (%)	34 (46.0)	33 (45.8)	40 (72.7)	*0.003*
Insulin, UI/die – median [IQR]	35 [16]	37 [18]	40 [16]	*0.095*
Lipid-lowering therapy – no. (%)	0 (0)	6 (8.3)	8 (14.6)	*0.006*
Anti-hypertensive therapy – no. (%)	29 (39.2)	41 (56.9)	38 (69.1)	*0.014*
ACEi/ARB – no. (%)	20 (27.0)	33 (45.8)	33 (60.0)	*0.001*
Beta-Blockers – no. (%)	1 (1.4)	4 (5.6)	4 (7.3)	*0.220*
Ca-Blockers – no. (%)	9 (12.2)	20 (27.8)	15 (27.2)	*0.060*
Alpha-Blockers – no. (%)	4 (5.4)	9 (12.5)	8 (14.6)	*0.220*

[a] $p<0.05$ *vs* No MVC; [b] $p<0.05$ *vs* 1 MVC.
* For the difference with the whole study cohort.
Abbreviations: ACEi, angiotensin converting enzyme inhibitors; ARB, angiotensin II receptors blockers; CAN, cardiac autonomic neuropathy; CKD, chronic kidney disease; DR; diabetic retinopathy; eGFR, estimated glomerular filtration rate; HDL, high-density lipoprotein; IQR, interquartile range; LDL, low-density lipoprotein; MVC, microvascular complication; mGFR, measured glomerular filtration rate.
